# Application of telesimulation in a medical undergraduate course during the SARS-CoV-2 pandemic: a quantitative and retrospective study

**DOI:** 10.1590/1516-3180.2021.0108.R2.22112021

**Published:** 2022-04-11

**Authors:** Carolina Felipe Soares Brandão, Gabriela Furst Vaccarezza, Regina Pose Albanese, Glória Celeste Vasconcelos Rosário Fernandes, Dario Cecilio-Fernandes

**Affiliations:** I BSc, DVM, MSc, PhD. Professor, Hospital Simulation, Medicine Program, Universidade Cidade de São Paulo (UNICID), São Paulo (SP), Brazil.; II DDS, MSc. Professor, Medicine Program, Universidade Municipal de São Caetano do Sul (USCS), São Caetano do Sul (SP), Brazil.; III MSc. Professor, Medicine Program, Universidade Municipal de São Caetano do Sul (USCS), São Caetano do Sul (SP), Brazil.; IV MD, PhD. Professor, Medicine Program, Universidade Cidade de São Paulo (UNICID), São Paulo (SP), Brazil.; V MSc, PhD. Researcher, Department of Medical Psychology and Psychiatry, School of Medical Sciences, Universidade Estadual de Campinas (UNICAMP), Campinas (SP), Brazil.

**Keywords:** Simulation training, Education, medical, undergraduate, COVID-19, Telesimulation, Medical education, Undergraduate, Coronavirus disease 19

## Abstract

**BACKGROUND:**

Because of the social isolation and distancing measures that were imposed to stop the spread of coronavirus disease 19 (COVID-19), new ways of teaching were implemented.

**OBJECTIVES:**

To describe the implementation of telesimulation and seek to assess students’ perceptions regarding telesimulation.

**DESIGN AND SETTING:**

Retrospective quantitative study conducted within the hospital simulation at a private medical school in São Paulo, Brazil.

**METHODS:**

After telesimulation training, students answered a questionnaire that provided an overall assessment of this activity, self-assessment and assessments of the facilitators and infrastructure provided by the University.

**RESULTS:**

Among the students, 50% reported that the activity was below expectations and 45% reported that it was in line with their expectations. The strong points of the activity were the clinical cases, workload and teachers. The main challenge was students’ difficulty in reflecting on their learning and the infrastructure.

**CONCLUSIONS:**

Since students have less experience and fewer clinical encounters than residents or professionals, they also face more difficulty. Although telesimulation may have provided a valid alternative to replace simulation training during the COVID-19 pandemic, more face-to-face activities should be offered to students, when possible.

## INTRODUCTION

Over recent years, the use of clinical simulation within healthcare education has grown, since it offers the opportunity to integrate skills and clinical reasoning with motor and behavioral skills simultaneously. Moreover, clinical simulation allows students to learn from their mistakes in a safe environment while replicating a real-life environment.^1,2^

The severe acute respiratory syndrome coronavirus 2 (SARS-CoV-2) pandemic has brought major disruption to all academic institutions, particularly medical courses. Within a short time, face-to-face activities were transformed into online activities mediated through technologies.^3^ Most activities focused on the theoretical part of the curriculum or, at most, clinical reasoning, since all in-service training was suspended, potentially bringing a significant loss to students’ learning, especially among those at the end of the course.

To reduce students’ losses, we implemented telesimulation, which is a model that has been used to provide education, training and evaluation within healthcare. Telesimulation is defined as a process that combines telecommunication and simulation, to allow most students to attend simulations online.^4,5^ Telesimulation has also been described as a useful resource in other fields such as robotic surgery and ophthalmological surgery,^4-8^ which do not particularly belong to emergency medicine. However, these are super-specialized fields and may not be applicable to undergraduates.

## OBJECTIVES

The aim of our study was to describe the implementation of telesimulation and seek to investigate medical students’ satisfaction with telesimulation.

## METHODS

The simulation took place every week between August and December 2020 since it is integrated into the institution’s competency-based curriculum. Students were divided into two groups, with an interval of one hour for sanitization procedures. Each week, there was a new opportunity for other students to participate as volunteers, which promoted the opportunity for everyone to participate.

The students in the face-to-face activity were volunteers, with a maximum of six students per activity. Before the start of every session, the students were guided through the biosecurity norms and signed an imaging rights statement, since the rest of the students were following the activity remotely and synchronously. A variety of scenarios were played out, around the following themes: chronic obstructive pulmonary disease, pulmonary thromboembolism, foreign body airway obstruction, pancreatitis, septic shock and diabetic ketoacidosis.

**Figure 1.**
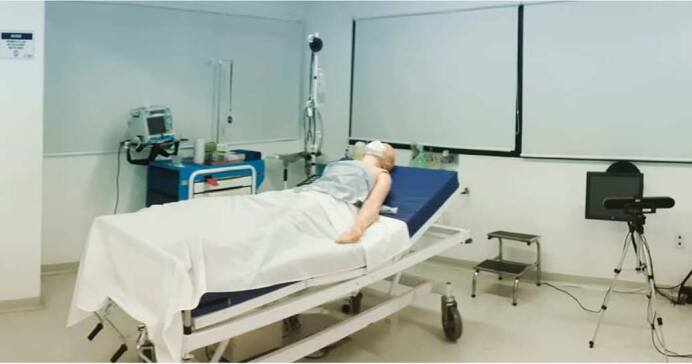
Example of a room for telesimulation.

Our hospital simulation consisted of a large room that had been adapted to follow all sanitary protocols. The simulation included cameras and microphones that allowed us to film and record the simulated patient (who could be either an actor or a simulator) and undertake multiparametric monitoring through the institution’s communication platform (Figure 1), which in this case was Microsoft Teams. In this model, one teacher stayed in the face-to-face simulation and another in the online environment attending to students who were participating remotely.

The technique used for these simulations consisted of a rapid cycle of deliberate practice, since those students lacked the necessary practice, which consequently would make it unlikely that there would be any reflection process. A rapid cycle of deliberate practice splits simulation scenarios into small segments with feedback, in the form of pause, feedback and try again. Students only move forward when no mistake is made in that segment.^9^ In addition, the input from the facilitator made the simulated experience more motivating for the students, especially those at home. A second facilitator was responsible for engaging and interacting with students at home. This form of telebriefing was performed considering that the students already were aware of the feedback structure that would be adopted in the rapid cycle of deliberate practice. Students could access the simulation session on their smartphones or other device. Another facilitator was responsible for the online students in case of doubts.

After the activities of that semester had been completed, all students received an online questionnaire, which it was not mandatory to respond to. This asked the students to give their overall assessment of this activity, self-assessment and assessment of facilitators and infrastructure provided by the university. The online questionnaire contained a mix of dichotomous questions (yes or no), gradings (ranging from 0 to 10), multiple-choice questions and open questions (Appendix 1).

This data collection was approved by our institution’s research ethics committee (protocol number 37360820.8.0000.0064) on September 9, 2020. Quantitative data were analyzed using descriptive analysis, and qualitative data were analyzed using content analysis, as proposed by Bardin.^10^ After categorizing the answers for each open-ended questions, we used descriptive analysis to present the results.

## RESULTS

Among the 180 students who were involved in this activity, only 11% (n = 20) answered the questionnaire. Out of those students, 18 participated in the scenario (face-to-face activity), one did not want to participate in the scenario and one wanted to participate but did not have the chance. All the students who answered the questionnaire considered simulation important in medical training and agreed that the clinical cases selected for the activities were good.

Concerning the implementation of telesimulation, 50% reported that it was below their expectations and 45% reported that it was in line with their experience (5% reported that it was above their expectations). Half of the students felt safe during the simulation and had a good experience and 10% did not like it. 30% of the students felt exposed, although they felt that it was a good experience. Most of the students considered that Microsoft Teams was an adequate tool. The students also reported that teachers were accessible (70%). The workload was considered adequate by 50% of the students; adequate but would have been better with more hours, by 30%; and inadequate and ought to have been extendezzzd for more hours, by 20%.

Although most of the students (85%) reported that telesimulation did not provide the possibility of leading to reflection in the same way as would occur with face-to-face simulation, most of them (70%) said that they would be willing to take some other course using telesimulation. Lastly, the qualitative analysis showed that the main barriers reported were the following: infrastructure (35%); applied methodology (25%); volunteers’ performance (20%); workload (10%); proposed scenarios (5%); and teachers (5%).

## DISCUSSION

Despite the students’ understanding about the pandemic and the teaching effort needed to adapt to simulated training, they showed great frustration regarding telesimulation. This was probably because simulation training is one of the activities most eagerly expected by students. It takes place while the students are still in the preclinical phase. This is the time at which clinical reasoning becomes integrated with procedural and behavioral conduct.

We identified some barriers that impeded the activity. Oscillations in internet connections, inconsistencies in using Microsoft Teams and difficulties in sound recording in the simulated environment hampered the students’ understanding. These barriers have also been reported elsewhere, especially in low and middle-income countries.^11^

One limitation of the present study was that the students who participated in the research were more likely to rate this activity positively, since most of them participated in the face-to-face simulation. Another limitation was that this study focused only on the students’ satisfaction without measuring their learning.

## CONCLUSION

Use of telesimulation has supported clinical training to some degree during the COVID-19 pandemic. Although telesimulation may provide a valid alternative for replacing simulation training during COVID-19, more face-to-face activities should be offered to students, when possible.

## Appendix 1. Medical student experience instrument for use of a new telesimulation-based educational tool during the SARS-CoV-2 pandemic.

(Start by reading and signing the free and informed consent statement. This is MANDATORY for continuing the process).

Date: __________

Group:

A. blue [ ] green [ ]

B. blue [ ] green [ ]

Gender:

Female    [ ]

Male     [ ]

Non-binary    [ ]

I don’t want to answer [ ]

Age (in years): _______________

Stage: _______________

Do you know of any other university institution that is carrying out telesimulation during this pandemic?

No [ ]

Yes [ ]

– Please write the name of the institution and its location, city, state and country: _______________

We ask you to answer all of the following items about your experience with the telesimulation training that was implemented. You must answer all items. Each item has only one valid answer. (Note: for some items, the responses are nested because they depend on the previous answer).

1. Do you consider simulation important for your undergraduate medical training?

Yes [ ]

No [ ]

Indifferent [ ]

2. Because of the pandemic, telesimulation was included as a curriculum subject. How has this experience been?

Above your expectations [ ]

Below your expectations [ ]

Indifferent [ ]

3. Have you volunteered for any in-service training?

No        [ ]

I wanted to but I couldn’t because of the pandemic [ ]

I didn’t ask for this method    [ ]

Yes        [ ]

In this telesimulation experience:

I felt exposed      [ ]

I did not like it      [ ]

I considered it valid     [ ]

I didn’t feel exposed. It was a good experience [ ]

4. Analysis on the process experienced:

Regarding the cases discussed, I considered that they:

Were very complex   [ ]

Could have been more complex [ ]

Were very simple   [ ]

Regarding the technological tool selected for telesimulation (Microsoft Teams), I considered that it was:

Suitable. No problem   [ ]

Poor. I would prefer another app [ ]

– State which app: _______________

Regarding the professor who facilitated the telesimulation/telefeedback, I considered that this person was:

Always accessible  [ ]

Not always accessible [ ]

Regarding the workload, I considered that:

It was good/adequate  [ ]

It should have been greater [ ]

It should have been smaller [ ]

It was poor/inadequate  [ ]

I considered that the infrastructure of the hospital simulation during the pandemic was:

Adequate  [ ]

Inadequate [ ]

Justify your response in a few words: _______________

5. Do you consider that telesimulation has contributed to your undergraduate medical training?

Yes [ ]

No [ ]

6. Do you consider that telesimulation can lead to reflection in the same way as occurs with face-to-face simulation?

Yes [ ]

No [ ]

7. Would you take another training course in telesimulation?

Yes [ ]

No [ ]

8. Check the factor that describes your biggest difficulty with this methodology:

The technology (which hampered my development) [ ]

The method (which did not favor my understanding) [ ]

The teacher (who did not facilitate the process) [ ]

9. In your opinion, what had the most positive impact on these telesimulation activities?

Workload   [ ]

Proposed scenario  [ ]

Infrastructure  [ ]

Professor   [ ]

Technology applied [ ]

Your performance  [ ]

10. In your opinion, what had the most negative impact on these telesimulation activities?

Workload [ ]

Proposed scenario [ ]

Infrastructure [ ]

Professor  [ ]

Technology applied [ ]

Your performance [ ]

11. Choose a score from 0 (worst) to 10 (best) for your overall assessment of the process that you experienced: _______________

12. Choose a score from 0 (worst) to 10 (best) for your self-assessment of this process: _______________

13. Describe, in one paragraph, your experience over the course of this process:

__________________________________________________________________________________________________________________________

__________________________________________________________________________________________________________________________

__________________________________________________________________________________________________________________________

Thank you for your participation in this process.
